# Functional characterization of zebrafish orthologs of the human Beta 3-Glucosyltransferase *B3GLCT* gene mutated in Peters Plus Syndrome

**DOI:** 10.1371/journal.pone.0184903

**Published:** 2017-09-19

**Authors:** Eric Weh, Hideyuki Takeuchi, Sanaa Muheisen, Robert S. Haltiwanger, Elena V. Semina

**Affiliations:** 1 Department of Pediatrics and Children’s Research Institute at the Medical College of Wisconsin, Milwaukee, Wisconsin, United States of America; 2 Department of Cell Biology, Neurobiology, and Anatomy at the Medical College of Wisconsin, Milwaukee, Wisconsin, United States of America; 3 Complex Carbohydrate Research Center, University of Georgia, Athens, Georgia, United States of America; 4 Department of Ophthalmology at the Medical College of Wisconsin, Milwaukee, Wisconsin, United States of America; Leibniz Institute on aging - Fritz Lipmann Institute (FLI), GERMANY

## Abstract

Peters Plus Syndrome (PPS) is a rare autosomal recessive disease characterized by ocular defects, short stature, brachydactyly, characteristic facial features, developmental delay and other highly variable systemic defects. Classic PPS is caused by loss-of-function mutations in the *B3GLCT* gene encoding for a β3-glucosyltransferase that catalyzes the attachment of glucose via a β1–3 glycosidic linkage to *O*-linked fucose on thrombospondin type 1 repeats (TSRs). B3GLCT was shown to participate in a non-canonical ER quality control mechanism; however, the exact molecular processes affected in PPS are not well understood. Here we report the identification and characterization of two zebrafish orthologs of the human *B3GLCT* gene, *b3glcta* and *b3glctb*. The *b3glcta* and *b3glctb* genes encode for 496-aa and 493-aa proteins with 65% and 57% identity to human B3GLCT, respectively. Expression studies demonstrate that both orthologs are widely expressed with strong presence in embryonic tissues affected in PPS. *In vitro* glucosylation assays demonstrated that extracts from wildtype embryos contain active b3glct enzyme capable of transferring glucose from UDP-glucose to an *O*-fucosylated TSR, indicating functional conservation with human B3GLCT. To determine the developmental role of the zebrafish genes, single and double *b3glct* knockouts were generated using TALEN-induced genome editing. Extracts from double homozygous *b3glct*^*-/-*^ embryos demonstrated complete loss of *in vitro* b3glct activity. Surprisingly, *b3glct*^*-/-*^ homozygous fish developed normally. Transcriptome analyses of head and trunk tissues of *b3glct*^*-/-*^ 24-hpf embryos identified 483 shared differentially regulated transcripts that may be involved in compensation for b3glct function in these embryos. The presented data show that both sequence and function of *B3GLCT/b3glct* genes is conserved in vertebrates. At the same time, complete *b3glct* deficiency in zebrafish appears to be inconsequential and possibly compensated for by a yet unknown mechanism.

## Introduction

Peters Plus Syndrome (PPS) is a rare autosomal recessive disease characterized by ocular defects, brachydactyly, short stature, characteristic facial features and developmental delay as well as other variable systemic defects affecting the skeletal, cardiovascular, genitourinary and central nervous systems [1–3]. The ocular defect most often associated with PPS is Peters anomaly (PA); however, other defects affecting the anterior segment of the eye have been reported [1–3]. PA is an adhesion between the cornea and the lens that results in a central corneal opacity, or leukoma, and can lead to blindness if not treated [4].

The causative gene for PPS was originally identified by Lesnik Oberstein in 2006 as *B3GLCT* (formerly *B3GALTL)* [5]. Since then, our and other groups have identified multiple *B3GLCT* mutations in patients with PPS and defined the phenotype associated with *B3GLCT* mutations [2, 5–15]. *B3GLCT* mutations result in classic PPS which is characterized by a triad of features including anterior segment defects (100%; Peters anomaly in 85%), short stature (100%), and brachydactyly (95%), as well as variable other highly penetrant features such as developmental delay (84%), congenital heart defects (40%), cleft lip/palate (37%) and other anomalies [3]. At the same time, PPS-like cases (often clinically diagnosed as PPS) which demonstrate overlapping phenotypes but lack one or more of the key triad of features seen in classic PPS were found to not be caused by mutations in *B3GLCT* [2, 3, 16–18].

Human *B3GLCT* is located on chromosome 13q12.3 and is divided into 15 exons encoding for a 498 amino acid protein. *B3GLCT* mRNA is expressed in a variety of different adult human tissues including heart, brain, lung, kidney, and skeletal muscle [19]. Based on sequence similarity and amino acid conservation at the catalytic site the encoded protein is predicted to follow the stereotypical domain architecture of other members of the GT31 glycosyltransferase family. The gene was initially described as encoding for a β1-3-galactosyltransferase based on sequence similarity [19], however this was revised once the catalytic activity of the enzyme was deduced [20, 21]. These studies demonstrated that B3GLCT is a β3-glucosyltransferase that catalyzes the attachment of glucose to *O*-linked fucose via a β1–3 glycosidic linkage on thrombospondin type 1 repeats (TSRs). TSRs can be found on over 60 human proteins, however only 49 of them contain the required consensus sequence to be fucosylated and subsequently modified by B3GLCT [22]. Proteins that have been shown to be modified by B3GLCT include Thrombospondin-1 [23], Thrombospondin-2 [24], Properdin [25], F-spondin [26], ADAMTS-13 [27], ADAMTSL-1 [28], and ADAMTS-5 [29]. Properdin has been shown to lack proper modification in patients with PPS [25] yet is still secreted, although at a somewhat lower amount than normal. Additionally, PPS patients do not display any features of Properdin deficiency, an X-linked immune disorder [30]. These data suggest that glucosylation by B3GLCT may not be necessary for the secretion and/or function of all possible targets of this enzyme.

B3GLCT was found to participate in a non-canonical ER quality control pathway by Vasudevan and colleagues [31]. Their report found that modification by B3GLCT marks properly folded TSR motifs and is necessary for proper secretion of a subset of TSR containing proteins. Still, the underlying molecular mechanisms leading to PPS through disruption of this pathway are not understood and animal models of *B3glct* deficiency are yet to be developed. In this manuscript we report the characterization of two zebrafish orthologs of the human *B3GLCT* gene: *b3glcta* and *b3glctb*. We show that both zebrafish orthologs are well conserved with the human B3GLCT gene/protein and strongly expressed throughout the developing zebrafish embryo. We also demonstrate a lack of a visible morphological phenotype in *b3glcta*^*-/-*^, *b3glctb*^*-/-*^ or *b3glcta*^*-/-*^*; b3glctb*^*-/-*^ genetic lines generated via TALEN-mediated genome editing. Further studies of a potential compensatory mechanism via transcriptome analyses identified a variety of differentially regulated genes which may be capable of compensating for the lack of *b3glct* during zebrafish development.

## Results

### Identification and characterization of the zebrafish *b3glcta* and *b3glctb*

**Fig 1:**
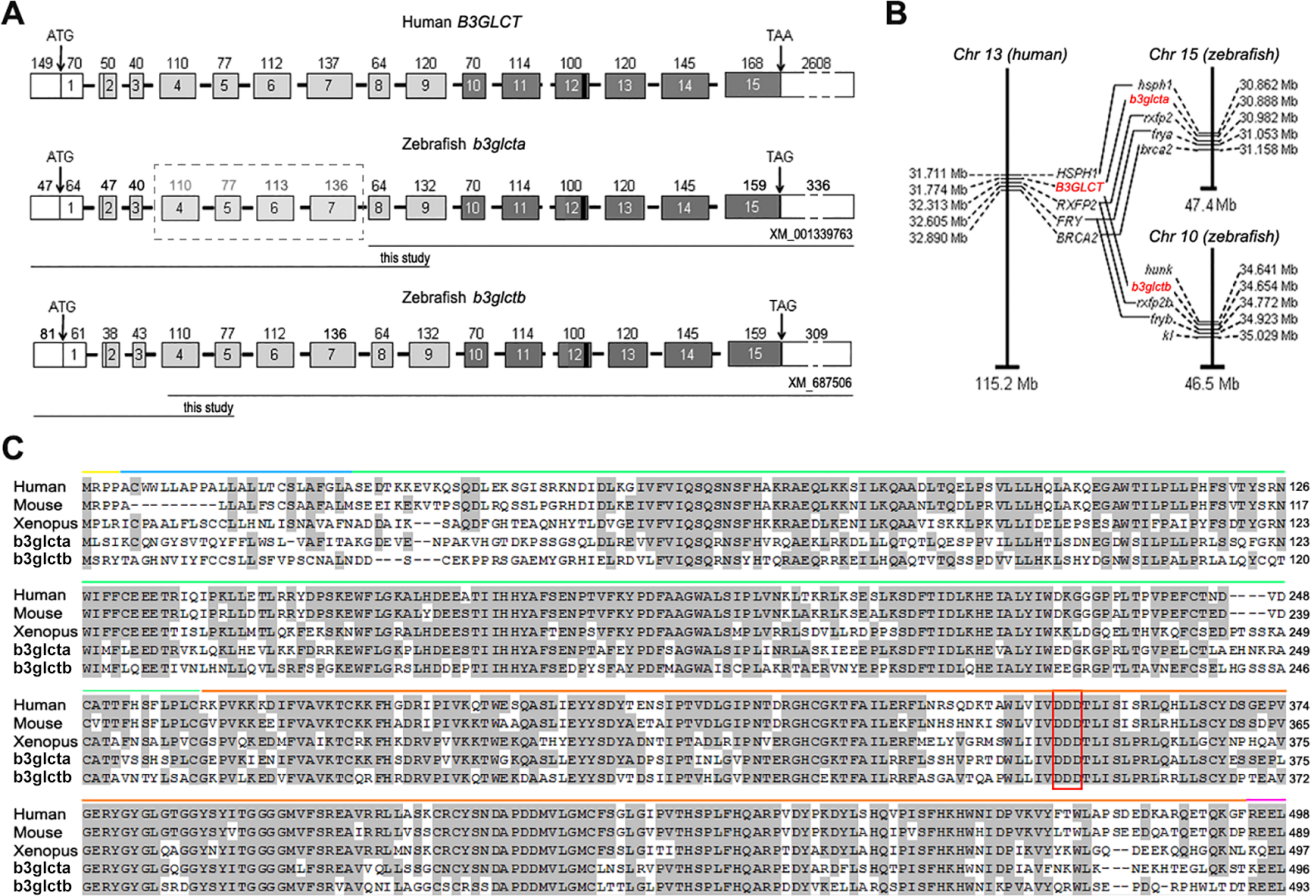
Exonic structure, genomic context and multiple species alignment of B3GLCT/b3glct. (A) The two zebrafish orthologs of B3GLCT show overall similar exonic arrangement. The number of each exon is located within each box and the size of the exon (in base pairs) is shown above each exon. The 5’ and 3’ UTRs are indicated preceding the first ATG and following the stop codon (TAA/TAG). White indicates the N-terminal signal sequence, light grey indicates the stem region and dark grey indicates the catalytic domain. The vertical black bar in exon 12 of each gene indicates the location of nucleotides encoding for the catalytic tri-aspartic acid residues. Horizontal lines underneath the zebrafish genes indicate previously annotated sequence and sequence identified in this study. (B) Schematic of genomic context for B3GLCT/b3glct. (C) Multiple species alignment of B3GLCT orthologs from human (NP_919299), mouse (NP_001074673), Xenopus (NP_001072551), and zebrafish. Blue bar indicates signal peptide, green indicates stem region and orange indicates catalytic core. Grey shading of amino acids indicates conservation. The DxD motif is boxed in red.

A search for zebrafish orthologs of the human *B3GLCT* gene (NP_919299.3) using NCBI BLASTp and zebrafish databases identified two different protein-coding sequences. The first putative zebrafish ortholog (*b3glcta*, XM_001339763.3) comprised a 1240-bp transcript predicted to encode for a protein of 300 amino acids in length (XP_001339799) and was located on chromosome 15. The second putative zebrafish ortholog (*b3glctb*, XM_687506.5) included a 1686-bp mRNA sequence predicted to encode for a protein of 452 amino acids in length (XP_692598.5) and was located on chromosome 10 (Fig 1). When the identified *b3glcta* and *b3glctb* sequences were aligned with the human *B3GLCT*, both zebrafish orthologs were found to likely be lacking several 5′ exons encoding for a large portion of their N-terminus and thus appeared to be incomplete (Fig 1A). To determine full-length transcripts for both genes, 5′-RLM RACE was performed and identified additional 5′ sequences (634-bp for *b3glcta* and 186-bp for *b3glctb*). The *b3glcta* and *b3glctb* full transcript sequences were verified by a series of independent PCRs (Fig 1A) and deposited to GenBank under the following accession numbers: *b3glcta*: NM_001302251.1, NP_001289180.1; *b3glctb*: NM_001302248.1, NP_001289177.1.

The resultant full-length transcript of *b3glcta* is 1874-bp long and is predicted to encode for a 496 amino acid peptide (Fig 1A and 1C). Similarly, the full-length transcript of *b3glctb* is 1872-bp in length and is predicted to encode for a 493 amino acid peptide (Fig 1A and 1C). Alignment of the complete predicted amino acid sequences of each ortholog with the human B3GLCT protein identified 65% and 57% identity for *b3glcta* and *b3glctb*, respectively. Multiple protein alignment using human, mouse, frog and zebrafish sequences demonstrated a high level of conservation for various B3GLCT/B3glct/b3glct proteins (Fig 1C). The N-terminus of B3GLCT, which contains the signal sequence, demonstrated the lowest level of homology while the predicted catalytic region showed the strongest conservation, especially at and around the predicted “DxD” nucleotide-binding domain [32] (Fig 1C). At the same time, predictions by SignalP and TargetP indicated that all examined orthologs should contain an ER targeting signal at the N-terminus despite the low level of conservation.

The genomic context for each *b3glct* gene was examined using the UCSC genome and Genomicus synteny browsers. The genomic region of *b3glcta* is syntenic with human *B3GLCT*, flanked by *hsph1*/*HSPH1* on the 5′ end and *rxfp2/RXFP2*, *frya/FRY*, and *brca2/BRCA2* on the 3′ end (Fig 1B). The genomic region of *b3glctb* is only partially syntenic with the human *B3GLCT* gene, sharing *rxfp2b/RRXFP2b* and *fryb/FRY*, but not *brca2/BRCA2*, on the 3′ end of the gene, and showing no synteny on the 5′ end.

### Expression of *b3glcta* and *b3glctb* during development

**Fig 2:**
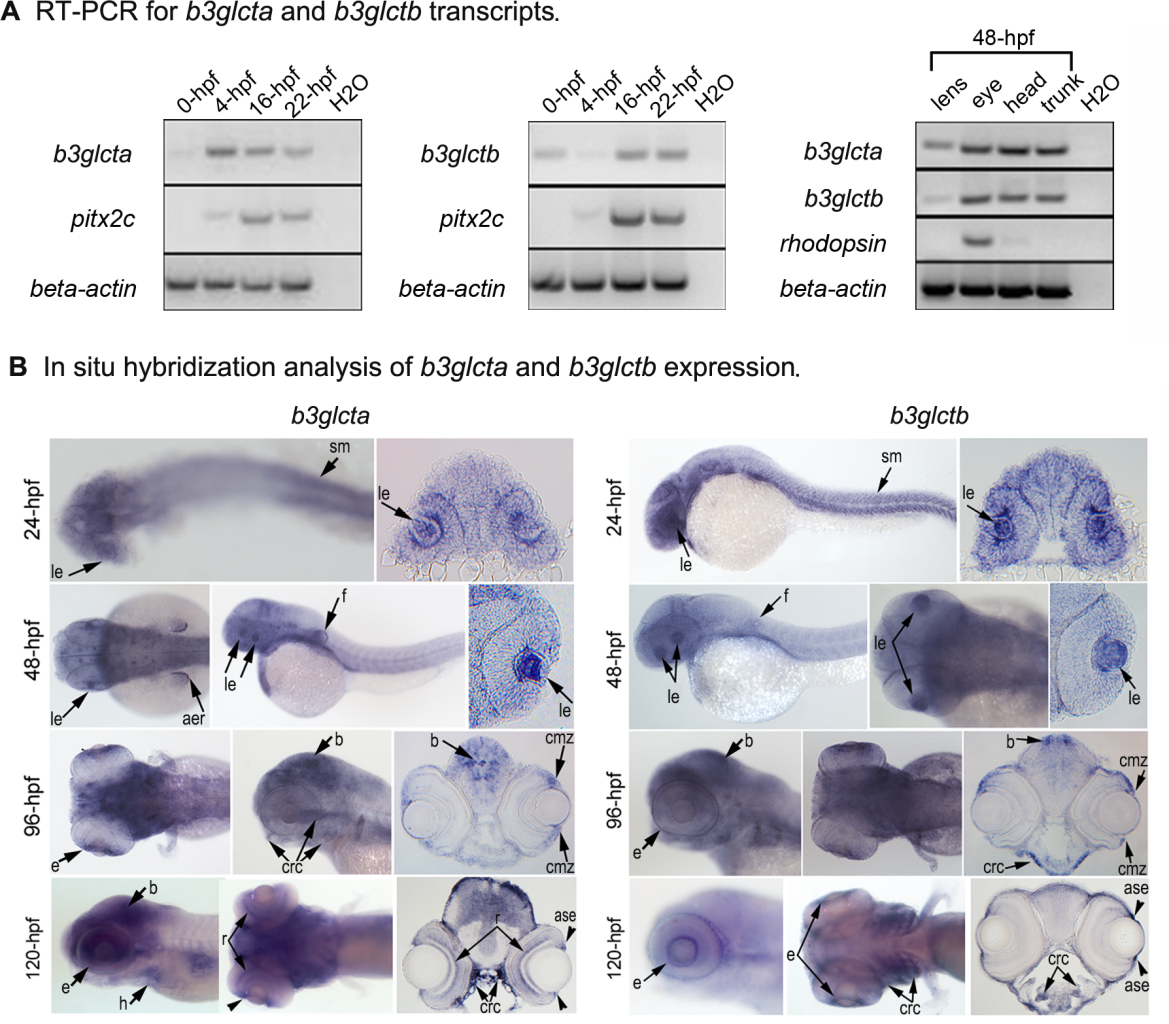
Embryonic expression of zebrafish *b3glct* genes. (A) RT-PCR analysis of *b3glct* expression demonstrates robust expression of both *b3glcta* (left panel) and *b3glctb* (middle panel) at different stages of development in whole embryos as well as various embryonic tissues at 48-hpf (right panel). Controls included *pitx2c* as negative control for 0-hpf, *rhodopsin* as negative control for the lens, *beta-actin* as positive control for all tissues and H_2_O as negative contamination control for all reactions. (B) In-situ hybridization analysis of *b3glcta* and *b3glctb* expression demonstrates broad expression in 24-120-hpf embryos with enrichment in the developing eyes, fins, brain, craniofacial region and somites. aer–apical ectodermal ridge, ase–anterior segment of the eye, b–brain, cmz–ciliary marginal zone, crc–craniofacial cartilage, e–eye, f–fins, h–heart, le–lens, sm–skeletal muscles.

We next investigated the spatio-temporal expression pattern of *b3glcta* and *b3glctb* to determine their possible role in embryonic development. mRNA collected from whole zebrafish embryos at 0-hpf, 4-hpf (onset of zygotic transcription), 16-hpf (lens placode) and 22-hpf (formation of the solid sphere lens mass) as well different tissues/regions of 48-hpf embryos (lens, eye, head and trunk) was analyzed (Fig 2A). We detected both *b3glct* transcripts at all developmental stages including 0-hpf, indicating that they are maternally provided. Also, expression of both *b3glct* genes was evident in all examined tissues of 48-hpf embryos (Fig 2A). The *pitx2c* gene was included as a control for 0-hpf (since it is not maternally contributed) [33], *rhodopsin* was used as a negative control for lens (expressed in retina only), and *beta-actin* as loading control for all time points.

To investigate the spatio-temporal expression of both genes, antisense and sense mRNA riboprobes specific to each *b3glct* transcript were generated and utilized for in situ hybridization in 24-120-hpf embryos. Consistent with the RT-PCR data presented above, hybridization with antisense probes for both transcripts identified broad expression patterns (Fig 2B); no specific stain was observed with the control sense probes (S2 Fig). At 24-hpf *b3glct* transcripts are expressed widely in the craniofacial region, brain and the developing somites. At 48-hpf *b3glct* transcripts continue to be detected in the brain, heart, fins, and somites and become more prevalent within the lens in the eye. At 96- and 120-hpf strong expression of both *b3glct* genes is observed in the developing eye, brain, pharyngeal arches, and heart. Broad zebrafish *b3glct* expression is consistent with both human and mouse *B3GLCT/B3glct* data (biogps.org).

### TALEN-based disruption of *b3glct* genes in zebrafish

**Fig 3:**
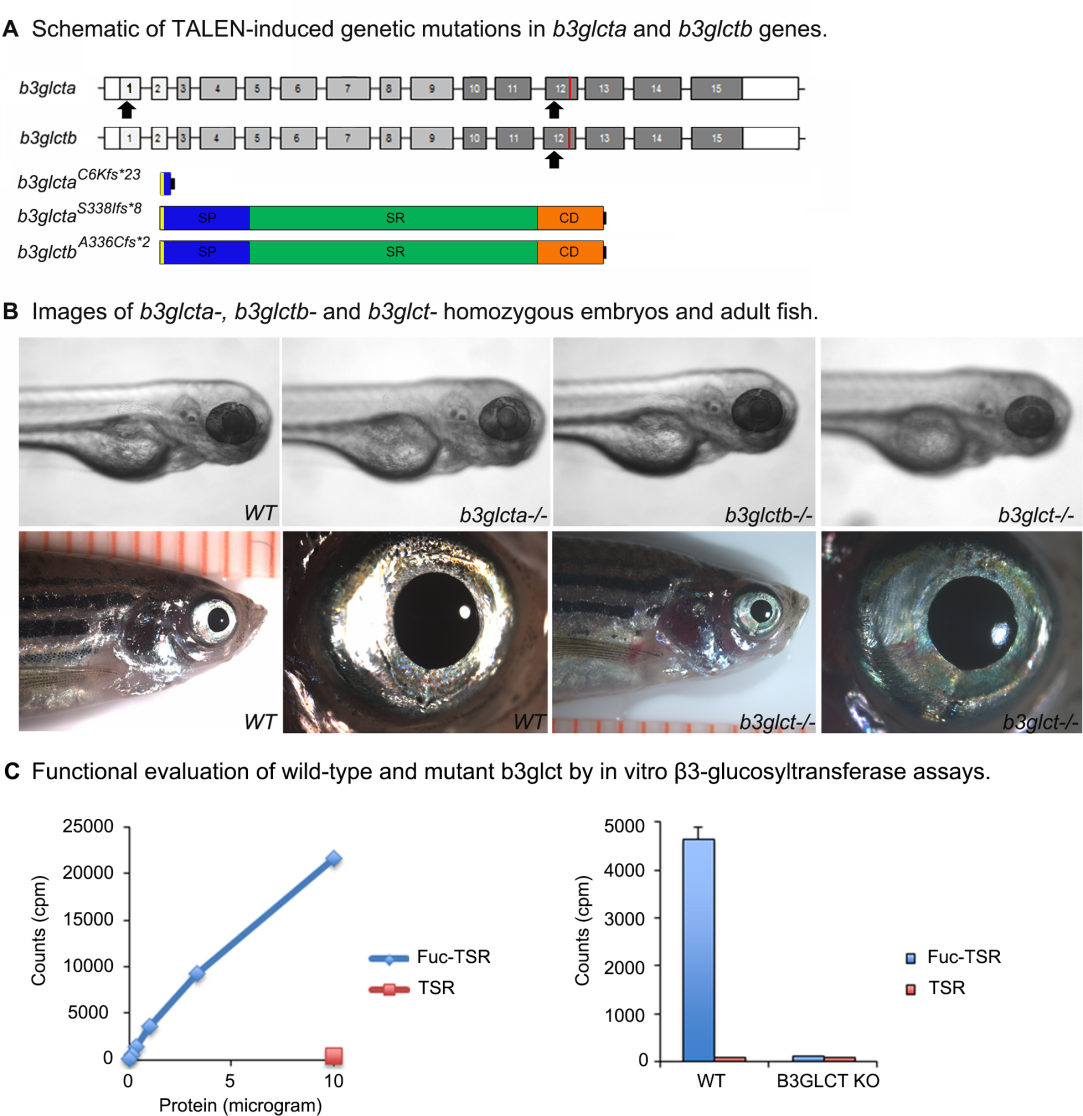
Genetic disruption of *b3glct*. (A) Schematic of *b3glct* genes indicating TALEN target sites (exon 1 and 12 for *b3glcta* and exon 12 for *b3glctb*, black arrows). The predicted protein product resulting from TALEN mediated disruption is shown. Editing events in the first exon of *b3glcta* are predicted to disrupt nearly the entire coding region of the transcript. For both *b3glcta* and *b3glctb*, editing in the 12^th^ exon is predicted to result in loss of most of the catalytic domain including the catalytic core and KDEL-like ER retention signal. Blue (SP)- Signal Peptide, Green (SR)- Stem Region, Orange (CD)- Catalytic Domain. (B) Images of zebrafish embryos at 5-dpf and adult zebrafish showing no gross morphological defects associated with loss of *b3glct*. (C) Functional evaluation of wild-type and mutant b3glct by *in vitro* β3-glucosyltransferase assays. Left panel- the endogenous β3-glucosyltransferase activity toward *O*-fucosylated TSR3 is dependent on the amount of protein in the wild type zebrafish homogenate; control reaction with 10 μg homogenate was performed using unmodified TSR3. Right panel- the endogenous β3-glucosyltransferase activity toward *O*-fucosylated TSR3 in the homogenate of double homozygous *b3glct* embryos is profoundly reduced compared with that in the wild type zebrafish homogenate; control reactions were performed using unmodified TSR3. Assays were performed in triplicate. Error bars indicate s.d.

To generate genomic mutations in the *b3glcta/b* genes, custom TALEN nucleases were designed to bind and create double stranded DNA breaks (DSBs) in specific *b3glcta* or *b3glctb* regions. The repair process of DSBs through the non-homologous end joining (NHEJ) pathway creates short insertions or deletions of nucleotides resulting in frameshift mutations that usually lead to loss of function due to truncation of the protein product. The first exon of *b3glcta* was initially targeted to create a complete loss of the protein product (Fig 3A). Custom TALE nucleases were designed by Cellectis and injected into single cell stage zebrafish embryos. These embryos were raised and germline founders were identified. Embryos derived from founder crosses were raised and genotyped to identify heterozygous carriers of frameshift mutations. Nine frameshift mutations were identified in these adult fish; all were predicted to result in a severe truncation of the protein product (S1 Table). Adult fish heterozygous for frameshift mutations were in-crossed to generate compound heterozygous embryos expected to have no functional *b3glcta*. The resultant embryos (n~800) were examined for an abnormal phenotype between 24- and 120- hpf and a subset of embryos (n = 70) was genotyped. The expected Mendelian ratios were observed indicating no embryonic lethality due to loss of *b3glcta*. No phenotype was identified which co-segregated with the *b3glcta* mutant allele. Embryos obtained from heterozygous in-crosses were raised to adulthood and compound heterozygous adults (*b3glcta*^*-/-*^) were identified in the expected ratios, indicating no lethality during the larval stage. To exclude a possible role of maternal transcript, *b3glcta*^*-/-*^ adults were in-crossed and produced ~400 embryos with no visible phenotype.

To further explore functional deficiency for *b3glct*, custom TALENs were created to disrupt either *b3glcta* or *b3glctb*, targeting the exons containing coding sequences for the catalytic domains of both enzymes (Fig 3A). This would ensure production of a truncated protein lacking the DxD motif in the catalytic domain, which is required for function of both enzymes. Therefore even if downstream initiation or alternative splicing are possible for the *b3glct* genes the resulting mutant alleles would ultimately produce non-functional protein products. This approach generated multiple mutant alleles (S1 Table). A single base pair (bp) deletion in *b3glcta* (c.1013delG, p.(S338Ifs*8)) and a 22-bp deletion in *b3glctb* (c.1005_1026del22, p.(A336Cfs*2)) were selected for further studies. These mutations were predicted to disrupt the catalytic domain of each enzyme and result in a truncated protein product lacking the C-terminal portion including the DxD motif and KDEL-like ER retention signal (Fig 3A). Adults carrying these mutations were identified and in-crossed to generate single homozygous knockouts (*b3glcta*^S338Ifs*8^ (*b3glcta*^*-/-*^) or *b3glctb*^A336Cfs*2^ (*b3glctb*^*-/-*^)) as well as double homozygous knockouts (*b3glcta*^S338Ifs*8^; *b3glctb*^A336Cfs*2^ (*b3glct*^*-/-*^)). No obvious developmental phenotype was observed in any embryos which co-segregated with loss of function mutations in either gene or the combination of both (Fig 3B). Embryos were raised to adulthood and genotyped. The expected Mendelian ratios were observed indicating no lethality during maturation of zebrafish deficient for *b3glct*. Adult zebrafish deficient for *b3glct* were examined for gross morphological defects, however none were identified (Fig 3B) and no changes in general locomotor activity or other gross behavioral changes were observed.

### Evaluation of wild-type and mutant b3glct activity

In order to confirm loss of function of enzymatic activity, *in vitro* glucosylation assays were performed. The *in vitro* glucosylation assay demonstrated that extracts from wildtype embryos contain active b3glct enzyme capable of transferring glucose from UDP-glucose to an *O*-fucosylated TSR, which indicates that the function of b3glct is conserved with human B3GLCT (Fig 3C, left panel). When the assay was repeated using extracts from double homozygous *b3glct*^*-/-*^ embryos, b3glct activity was less than 1% of wildtype extracts, demonstrating that these mutant animals lack active b3glct (Fig 3C, right panel).

### Transcriptome analysis of *b3glct* mutants

Microarray analysis revealed 1059 differentially regulated probes in the head tissue of *b3glct*^*-/-*^embryos and 1232 differentially regulated probes in the trunks of *b3glct*^*-/-*^ embryos compared to wild-type. Of these, 483 differentially regulated probes were shared between both the head and trunk tissues (S1 Fig, S2 Table). All but one (*mamdc2b*) of these genes were differentially expressed in the same direction in both tissues. Of these probes, 198 mapped to at least one orthologous gene in humans, 160 of which were unique genes. Since b3glct is part of a non-canonical ER quality control pathway we searched for genes on this list which participate in ER quality control or are related to ER function. Manual curation determined that 18 of these genes have functions related to ER homeostasis, quality control or the unfolded protein response (Table 1).

**Table 1:** Summary of differentially regulated genes implicated in ER quality control, unfolded protein response or cell survival.

Fold Change *b3glct*^*-/-*^		Log2FC *b3glct*^*-/-*^		ZebrafishGene	Human Ortholog	Potential compensatory function
Head	Trunk	Head	Trunk			
9.85	3.16	3.30	1.66	*ahsa1a*	*AHSA1* (activator of HSP90 ATPase activity 1) [34]	Activates HSP90
3.44	4.58	1.78	2.20	*alg5*	*ALG5* (dolichyl-phosphate beta-glucosyltransferase) [35]	Participates in N-glycosylation pathway
3.24	3.11	1.70	1.64	*angel1*	*ANGEL1* (angel homolog 1) [36]	Interacts with translation initiation factor, ER-localized
2.97	2.96	1.57	1.57	*Aplnra*	*APLNR* (apelin receptor) [37]	May modulate proteasome function, ER stress
2.87	2.64	1.52	1.40	*cast*	*CAST* (calpastatin) [38]	Inhibits calpains (proteases), may down regulate ER stress response when overexpressed
2.68	2.31	1.42	1.21	*eef2l2*	*EEF2* (eukaryotic translation elongation factor 2) [39]	Phosphorylation results in reduced translation during unfolded protein response
2.49	2.59	1.31	1.37	*eif1axa*	*EIF1AX* (eukaryotic translation initiation factor 1A, X-linked) [40]	Translation factor, down regulation reduces protein synthesis
2.19	2.42	1.13	1.28	*faima*	*FAIM* (Fas apoptotic inhibitory molecule) [41]	Inhibits cell death
0.50	0.51	-1.00	-0.98	*ficd*	*FICD* (FIC domain containing) [42]	Reversibly activates and inactivates BIP (HSPA5)
0.49	0.33	-1.04	-1.62	*ier3ip1*	*IER3IP1* (immediate early response 3 interacting protein 1) [43]	Implicated in ER stress response
0.41	0.39	-1.29	-1.34	*lman2l*	*LMAN2L* (lectin, mannose binding 2 like) [44]	Interacts with ERGIC-53 to regulate glycoprotein export from the ER
0.41	0.39	-1.30	-1.36	*ltn1*	*LTN1* (listerin E3 ubiquitin protein ligase 1) [45]	Part of ribosome quality control mechanism, clears stalled ribosomes, allows for new proteins to enter the ER
0.36	0.33	-1.49	-1.58	*mif*	*MIF* (macrophage migration inhibitory factor) [46]	Appears to be chaperone protein for SOD1, downregulation results in increased accumulation of mutant SOD1 in ER
0.36	0.29	-1.49	-1.79	*nlrb5*	*NLRP1/NLRP3* (NLR family pyrin domain containing 1/3) [47, 48]	Upregulated during the ER stress response, mediated by ATF4
0.32	0.29	-1.65	-1.79	*ramp1*	*RAMP1* (receptor activity modifying protein 1) [49]	Important for delivering cargo from ER to cell surface
0.24	0.27	-2.04	-1.89	*serpinh1a*	*SERPINH1* (serpin family H member 1) [50]	Collagen specific chaperone protein
0.15	0.13	-2.78	-2.92	*slc37a4b*	*SLC37A4* (solute carrier family 37 member 4) [51]	Transports glucose-6-phosphate into the ER

## Discussion

Peters Plus syndrome is a highly variable disease characterized by multiple congenital abnormalities including anterior segment dysgenesis (Peters anomaly), short stature, brachydactyly, craniofacial dysmorphism, developmental delay and others. PPS is caused by loss-of-function mutations in the *B3GLCT* gene, which encodes for a protein that catalyzes the attachment of the terminal glucose of a disaccharide found on Thrombospondin Type 1 Repeats (TSR); the majority of *B3GLCT* pathogenic alleles are whole gene deletions or frameshift/nonsense variants with a few missense mutations that affect the highly conserved catalytic domain. B3GLCT is predicted to modify 49 TSR-containing proteins of varying function, some of which, when disrupted, cause human disease with overlapping features: *ADAMTS10*, *ADAMTSL2*, *ADAMTSL4*, *ADAMTS2*, *ADAMTS17* and *ADAMTS18* [52–57]. The initial attachment of fucose to the TSR motif has been shown to be essential for embryonic development as *Pofut2* knockout mice do not progress past gastrulation [22]. It has also been identified that the fucose attachment on TSRs is essential for proper protein secretion [27, 28].

POFUT2 and B3GLCT were recently shown to be part of a non-canonical ER quality control mechanism [31]. Both enzymes are localized to the ER where proteins in the secretory pathway fold, and POFUT2 has the unique ability to distinguish between folded and unfolded TSRs [58, 59]. Vasudevan and colleagues demonstrated that POFUT2 and B3GLCT modify target proteins while they fold in the ER. Sequential addition of the fucose and glucose by POFUT2 and B3GLCT, respectively, appears to mark properly folded TSRs. Reduction of POFUT2 using shRNA in cell-based assays results in secretion defects of all targets tested, while reduction in B3GLCT affects secretion of some targets but not others. For instance, B3GLCT knockdown reduces secretion of ADAMTSL2 but not ADAMTS13. Patients with PPS lack proper glucosylation on TSR containing proteins [25]; however, they do not display features associated with a total loss of function of many proteins modified by B3GLCT, e.g. PPS patients do not display ADAMTS13 deficiency (Thrombotic thrombocytopenic purpura). These data suggest that modification by B3GLCT is essential for proper protein folding/secretion or function of some targets but not others.

In this study we have identified two full-length orthologs of *B3GLCT* in the zebrafish. Both orthologs demonstrated strong conservation at the levels of nucleotide and protein sequence as well as genomic organization; additionally both genes are positioned in regions that show synteny with the human *B3GLCT* gene (with the genomic context around *b3glcta* being more preserved). The domain organization of the predicted zebrafish b3glct proteins was found to maintain the typical structure associated with proteins belonging to the GT31 family of glycosyltransferases: N-terminal signal sequence, stem region, catalytic domain and finally a KDEL-like retention signal. The N-terminal sequence appears to be the most variable between species; however, all orthologs are predicted to contain a signal sequence in the examined species, consistent with the fact that proper localization of B3GLCT/b3glct to the ER is required for correct function of this enzyme. The catalytic domain shows the most significant conservation, consistent with its functional importance and suggesting preservation of B3GLCT/b3glct activities in different species. The GT31 family is characterized by a ‘DxD’ motif surrounded by five motifs containing 12 invariable amino acid residues [19]. Both zebrafish orthologs are 100% conserved at the ‘DxD’ motif, the 12 conserved residues and the five structural motifs. In addition, three residues which when mutated have been found to cause PPS, p.(Asp349Asn), p.(Gly394Glu) and p.(Gly393Glu) [3, 16], are conserved in the zebrafish orthologs, further supporting functional conservation between the zebrafish and human b3glct/B3GLCT. Finally, functional studies presented in this report that utilized lysates of whole embryos produced by wildtype and double homozygous parents demonstrated that wildtype samples are capable of transferring glucose to an *O*-fucosylated TSR while lysates from *b3glct*^*-/-*^ double homozygous embryos are deficient in performing the same modification. Expression analysis of the zebrafish orthologs demonstrates a robust embryonic expression pattern with enrichments in tissues affected in PPS. Taken together, these data indicate that the function of zebrafish *b3glct* genes is conserved with the orthologous human gene.

The phenotypic data from the double homozygous loss-of-function *b3glct* lines did not indicate any significant role for the *b3glct* genes in the development of the zebrafish embryo. Functional assessment showed that no other enzyme compensates for b3glct-deficiency in zebrafish and thus the possibility that another protein performs this function in *b3glct* double mutants is unlikely. In this case, the absence of embryonic phenotype in *b3glct*-deficient zebrafish lines may be explained by the fact that, unlike in humans, the modification catalyzed by this enzyme has no functional significance for folding/secretion/function of its targets. At the same time, the strong conservation at the evolutionary and functional level argues that these enzymes should be important for normal zebrafish physiology. The observed difference in phenotypic outcomes between humans and zebrafish with complete B3GLCT/b3glct deficiency may be due to an unknown compensation mechanism.

We hypothesized that possible compensation pathways to overcome b3glct deficiency may involve specific TSR-containing proteins or general ER function. For TSR-containing proteins that are dependent on normal b3glct function for their proper secretion, an increase in their expression may help to supply the required amount to the developing tissues. There are 49 proteins with the required consensus sequence for *O*-fucosylation and subsequent glucosylation by B3GLCT; zebrafish orthologs for the majority of these genes, including several verified targets of B3GLCT (THBS1, ADAMTSL2, ADAMTS9), have been identified with the consensus sequence being conserved. Of particular interest are the proteins belonging to the large family of multi-domain extracellular protease enzymes, ADAMTS, which have been implicated in human disease with overlapping features of PPS as discussed above. Orthologs of all *ADAMTS* genes except for three (*ADAMTS4*, *ADAMTS19* and *ADAMTSL1*) are present in the zebrafish genome. The microarray data generated in this study did not reveal any change in regulation of any of the genes coding for proteins predicted to be modified by b3glct except for *c6* (upregulated in both tissues) and *rspo1* (downregulated in both tissues). The human diseases associated with each of these genes do not present with any features which overlap with PPS, making it unlikely that changes in the expression of these genes is capable of rescuing the loss of function of b3glct [60, 61]. Since the remaining putative targets of b3glct do not appear to be undergoing any changes in their expression, it is likely that they are being secreted normally and fulfilling their various functions during development.

Also, as discussed earlier, B3GLCT has been shown to be part of a non-canonical ER quality control system. ER quality control ensures that proteins which enter the endoplasmic reticulum are properly folded and modified before they can be secreted (reviewed in [62]). This pathway allows for the reverse translocation of unfolded or improperly modified proteins out of the ER and into the cytosol where they can be degraded. If the amount of unfolded protein exceeds the capability of the cell to degrade protein then the cell will initiate the unfolded protein response (UPR). One mechanism of the UPR is to upregulate ER chaperone proteins in an attempt to increase the ratio of folded proteins. If the unfolded protein load cannot be resolved than the cell can undergo apoptosis. Zebrafish may be able to compensate for the lack of properly modified TSR domains through some as of yet unknown mechanism which may help to overcome the genetic loss of *b3glct*. Our microarray analysis revealed 483 differentially regulated probes in both the head and trunk tissues of double homozygous embryos compared to wild-type. It is not immediately clear which, if any, of these genes may be able to compensate for the loss of *b3glct*. Interestingly, one of these shared genes, *ahsa1a*, has been shown to act as an ATPase of the chaperone protein Hsp90 in humans [34]. Hsp90 is a general chaperone within the ER and an upregulation of the ATPase associated protein *ahsa1a* indicates that there may be an increase in unfolded proteins since ATP hydrolysis is essential for the chaperone function of Hsp90 [63]. At the same time these data do not provide evidence of ER stress or an unfolded protein response since none of the hallmark genes of the UPR appear to be upregulated. Interestingly, a related pathway (O-fucosylation of EGF repeats) was shown to be temperature-sensitive in Drosophila mutant strains [64]; a similar possibility may need to be further investigated in *b3glct*-deficient lines. Additional studies of the outcomes of b3glct deficiency in zebrafish may provide insight into disease mechanisms and/or suggest potential modifiers of Peters Plus Syndrome in humans.

## Conclusions

Our data suggest that the zebrafish orthologs of B3GLCT show significant sequence and functional conservation but have no apparent effect on normal zebrafish development when disrupted by genetic mutations. Preliminary transcriptome analysis has revealed changes in gene expression which may compensate for b3glct deficiency in *b3glct*^*-/-*^ zebrafish embryos. Further studies also examining changes in the proteome of these embryos may yield more clues to determine how zebrafish respond to/compensate for the lack of b3glct. These insights could lead to better understanding of the mechanisms underlying human PPS, its variability, and even possibly result in the development of novel therapeutic approaches for treatment of PPS and related disorders.

## Materials and methods

### Animals

Zebrafish, *Danio Rerio*, were maintained in a 14/10 hour light/dark cycle. The embryos were raised at 28.5°C in E2 medium containing phenylthiourea (PTU) to inhibit pigment formation and extend the period of transparency of the fish during development. The developmental stage of each embryo was assessed by previously described morphological criteria [65]. Experimental procedures were conducted with approval from the animal care and use committee at the Medical College of Wisconsin.

### Identification and characterization of zebrafish *b3glcta* and *b3glctb* genes

mRNA was isolated from 24 hours post fertilization (hpf) embryos using TRI Reagent® (Sigma-Aldrich, St. Louis, MO, USA) following the manufacturer’s supplied protocol. Purified mRNA was assessed for quality/quantity using non-denaturing agarose gel electrophoresis and spectrophotometry using a Nanodrop 1000 (Nanodrop Products, Wilmington, DE, USA) before processing with the RLM-RACE kit. Ambion’s FirstChoice® RLM-RACE Kit (Thermo Fisher Scientific, Waltham, MA, USA) was used to amplify transcripts with intact 5’ cap using gene-specific primers and kit reagents. Gene specific primers for each transcript, *b3glcta* and *b3glctb*, were designed based on manufacturer’s recommendations. The primers used for 5’ RACE are summarized in S3 Table. PCR amplicons were gel purified using the QIAGEN QIAquick® Gel Extraction Kit (QIAGEN, Valencia, CA, USA). The purified amplicons were then ligated into pCR^®^II-TOPO® (Thermo Fisher Scientific, Waltham, MA, USA) vectors following manufacturer’s protocols. The ligation products were then transformed into MAX Efficiency® DH5α™ Competent Cells (Thermo Fisher Scientific, Waltham, MA, USA) following standard procedures. Eight colonies for each transformation were selected and plasmid DNA was purified using the Invitrogen Mini Prep kit (Thermo Fisher Scientific, Waltham, MA, USA). The sequence of each insert was verified by Sanger sequencing using the M13F and M13R primers. The full sequences for both transcripts were submitted to GenBank under the accession numbers KJ680289 for *b3glct*a and KJ680290 for *b3glctb*.

Amino acid conservation was assessed using NCBI BLASTp (http://blast.ncbi.nlm.nih.gov/Blast.cgi) and PRALINE (http://www.ibi.vu.nl/programs/pralinewww/). The predicted protein sequences of the two zebrafish orthologs were aligned and compared for amino acid conservation with the human (*Homo sapiens*, NP_919299), mouse (*Mus musculus*, NP_001074673) and frog (*Xenopus tropicalis*, NP_001072551) B3GLCT/B3glct proteins. The signal and targeting peptide were predicted using SignalP (http://www.cbs.dtu.dk/services/SignalP/) and TargetP (http://www.cbs.dtu.dk/services/TargetP/). The genomic structure, location and surrounding region for both orthologs was analyzed using nucleotide BLAST (http://blast.ncbi.nlm.nih.gov/Blast.cgi), the UCSC Genome Browser (http://genome.ucsc.edu/) and the Genomicus Synteny browser (http://www.genomicus.biologie.ens.fr/genomicus-78.01/cgi-bin/search.pl).

### mRNA expression studies

The mRNA expression of both orthologs was analyzed using RT-PCR and in situ hybridization. For RT-PCR analysis, total RNA was extracted from whole wild-type embryos as well as from select tissues such as the lens, eye, head and trunk at different embryonic stages (0-48-hpf) using TRI Reagent® (Sigma-Aldrich, St. Louis, MO, USA). 0.5–1 μg of total RNA was used for cDNA synthesis with the SuperScript III cDNA synthesis kit (Thermo Fisher Scientific, Waltham, MA, USA). 1 μL of cDNA was used as a template for PCR with primers and conditions summarized in S3 Table. For in situ probes, partial sequences of each ortholog were generated by PCR using the primers listed in S3 Table; the resultant fragments were cloned into pCR^®^II-TOPO® plasmid (Thermo Fisher Scientific, Waltham, MA, USA) and verified by Sanger sequencing as described above. In situ hybridization was performed using specific antisense riboprobes generated from the obtained plasmids following previously described protocols [66]; additional control experiments included in situ hybridization that utilized sense probes (S2 Fig).

### Generation of *b3glcta* and *b3glctb* mutant lines

Targeted disruption of *b3glcta* and *b3glctb* was achieved through the use of TALEN (Transcription Activator-Like Effector Nuclease) technology reviewed by Kim and Kim [67]. Briefly, custom nucleases were designed to bind and create double stranded DNA breaks (DSBs) specifically in a region of the *b3glcta* or *b3glctb* gene. Two target sites were selected for *b3glcta* (exon 1 and exon 12) and one target site for *b3glctb* (exon 12). The TALEN pair targeting exon 1 of *b3glcta* was designed and synthesized by Cellectis (New Brighton, MN, USA). Target sites for exon 12 of *b3glcta or b3glctb* were chosen using the TALEN targeter software (https://tale-nt.cac.cornell.edu/node/add/talen) with the following custom parameters: minimum spacer length = 15, maximum spacer length = 20, minimum/maximum repeat array length = 19, G substitute-NN [68, 69]. TALEN binding locations were chosen so that the spacer region contained a unique restriction site to facilitate identification of editing events. TALE repeats were assembled using the Golden Gate method outlined by Sanjana [70] and TALE toolbox kit (#1000000019) from Addgene (Cambridge, MA, USA). Briefly, TALE repeats are assembled, in order, into a TALEN backbone vector using repeated rounds of restriction digestion and ligation. The assembled TALEN plasmid is then transformed into Max Efficiency DH5α competent cells (Thermo Fisher Scientific, Waltham, MA, USA) and plated onto LB agar plates containing 100 μg mL^-1^ carbenicillin. 15 colonies for each TALEN half were selected and grown overnight in LB media containing 100 μg/mL^-1^ carbenicillin. Plasmid DNA was extracted using the PureLink® Quick Plasmid Miniprep Kit (Thermo Fisher Scientific, Waltham, MA, USA). Plasmids were sequenced using primers outlined by Sanjana [70] and plasmids which contained correctly assembled TALE repeats were used as template for mRNA synthesis and subsequent injection into zebrafish embryos.

### mRNA synthesis

mRNA for injection into single cell stage zebrafish embryos was synthesized using the mMessage mMachine kit from Ambion (Thermo Fisher Scientific, Waltham, MA, USA) following the manufacturer’s supplied protocol. The plasmids encoding for the TALEN set synthesized by Cellectis were linearized using HindIII (New England Biolabs, Ipswich, MA, USA); the linearized plasmid was purified and used as a template for mRNA synthesis using the T7 RNA polymerase kit. The plasmids encoding for TALENs targeting exon 12 of *b3glcta* or *b3glctb* were digested using SmaI (New England Biolabs, Ipswich, MA, USA); the linearized plasmids were purified and used as a template for mRNA synthesis using the T7 RNA polymerase. The mRNA encoding for each TALEN was further processed to add a polyA tail using the Poly(A) Tailing kit from Ambion (Thermo Fisher Scientific, Waltham, MA, USA) to increase stability and enhance translation. All RNA was analyzed for integrity using gel electrophoresis and quantitated using a Nanodrop 1000 (Nanodrop Products, Wilmington, DE, USA).

### Generation of *b3glct* deficient zebrafish lines

Zebrafish injections were performed using a Drummond Nanoject II (Drummond Scientific, Broomall, PA, USA). 75 pg of each TALEN half for the Cellectis TALENs or 100 pg of each TALEN half for TALENs created in house were injected into each zebrafish embryo at the 1–4 cell stage. Total injection volume was equal to 9.2 nL and the solution was injected into the yolk of the embryo. Injected embryos were raised to adulthood (approximately 2 months of age) and in-crossed in a pairwise fashion. Embryos were collected from each crossing and genomic DNA was extracted using the previously published protocol [71]]. Briefly, individual embryos were placed in a PCR tube and 20 uL of 50 mM NaOH was added. The tube was heated to 95°C for approximately 15 minutes, then cooled to 4°C, and 1/10^th^ volume of 1 M Tris-HCl pH 8.0 was added. The samples were then vortexed and centrifuged to pellet any remaining tissues. 1 uL of the resultant supernatant was used for PCR amplification and subsequent restriction digestion. The targeted exons were amplified using the primers listed in S3 Table and Taq DNA polymerase from 5Prime (5 Prime GmbH, Hilden, Germany). The PCR amplicons were then digested with corresponding restriction enzymes and subjected to agarose gel electrophoresis to screen for genome editing events. BciVI, PmlI and NcoI were used for *b3glcta* exon 1, *b3glcta* exon 12 and *b3glctb* exon 12 respectively. A successful cutting event repaired using non-homologous end joining was expected to interrupt the restriction site and change the banding pattern. PCR products showing abnormal restriction patterns were sequenced bidirectionally on an ABI 3730xl sequencer using Big Dye Terminator v3.1 to determine the exact nucleotide mutation and predicted protein change. Founders which carried frameshift mutations were identified (generation P1) and crossed to generate embryos carrying frameshift mutations. These embryos were raised to adulthood (generation F1). The F1 adult fish were fin clipped and genomic DNA was extracted as above. Heterozygous adults carrying frameshift mutations were out-crossed to wildtype fish (generation F2). The F2 heterozygous adult fish were identified and in-crossed to generate homozygous mutant embryos. These embryos were analyzed for gross morphological defects using light microscopy. Double homozygous (*b3glcta*^*-/-*^;*b3glctb*^*-/-*^ or *b3glct*^*-/-*^) fish were generated by crossing single homozygous adults to create *b3glcta*^+/-^;*b3glctb*^+/-^ adults. These adults were in-crossed and the resultant embryos were analyzed for abnormal phenotypes using light microscopy. All embryos were raised to adulthood and adult double homozygous fish were identified as outlined above and crossed again to generate/evaluate fish with complete *b3glct* deficiency (including both maternal and zygotic transcripts) All generated mutations and lines are listed in S1 Table.

### In vitro assay of B3GLCT activity

Tissues from wild-type and *b3glct*^*-/-*^ 3-dpf larvae were homogenized in Tris-buffered saline, pH 7.4, containing Complete protease inhibitor cocktail (Roche, Basel, Switzerland) using a Polytron homogenizer. The homogenates were incubated on ice for 1 hour and centrifuged at 4°C for 20 min. Protein content in the supernatants was determined using BCA Protein assay (Thermo Fisher Scientific, Waltham, MA, USA) using BSA as a standard. Twenty μl standard enzymatic reaction mixtures contained 20 μM *O*-fucosylated TSR3 from human Thrombospondin1 [31] or unmodified TSR3, 0.2 μCi UDP-[6-^3^H]glucose (60 Ci mmol^-1^, American Radiolabelled Chemicals), 0.5% Nonidet P-40, 1 μg of embryo extract, in 50 mM HEPES, pH 6.8, 10 mM MnCl_2_. The reaction was incubated at 37°C for 2 hours and stopped by adding 900 μL of 100 mM EDTA pH 8.0. The samples were loaded onto a C18 cartridge (100 mg, Agilent Technologies, Santa Clara, CA, USA). After the cartridge was washed with 5 mL of water, the TSR proteins were eluted with 1 mL of 80% methanol. Incorporation of [6-^3^H]glucose into the TSR proteins was determined by scintillation counting of the eluate mixed with 4 mL of ScintiSafe Plus^TM^ (Thermo Fisher Scientific, Waltham, MA, USA) using a Liquid Scintillation Analyzer Tri-Carb 2910TR (PerkinElmer, Waltham, MA, USA). Reactions with unmodified TSR protein were used as negative control.

### Microarray analysis of gene expression in *b3glct* mutant tissues

Wildtype or *b3glct*^*-/-*^ double mutant 24-hpf embryos were dissected to obtain RNA from the head and trunk separately. Three independent pairs of *b3glct*^*-/-*^ double mutant fish were crossed to produce embryos. Tissues from 50 embryos per cross were combined for each collection and these three biological replicates were used for analysis. Tissue was solubilized in Tri-Reagent (Zymo Research, Irvine, CA, USA). RNA was subsequently extracted using the Direct-Zol RNA MiniPrep Plus Kit (Zymo Research, Irvine, CA, USA) following the manufacturer’s supplied protocol. RNA was eluted in 30μL of RNAse/DNAse free H_2_O. Each RNA sample was treated with DNAse I, Amplification Grade (Thermo Fisher Scientific, Waltham, MA, USA) following the manufactures instructions. Finally, DNAse treated RNA was purified using the RNA Clean and Concentrate Kit (Zymo Research, Irvine, CA, USA). Samples were eluted in 15μL of RNAse/DNAse free H_2_O. Purified RNA was sent to OakLabs for analysis using the ArrayXS Zebrafish V1 microarray (OakLabs, Hennigsdorf, Deutschland). This array assesses more than 60,000 targets based off of the Ensembl Zv9 release 75 of the zebrafish genome with each target having up to 20 representative probes on the array. RNA was checked for integrity using an RNA 600 Pico Kit on a 2100 Bioanalzer (Agilent Technologies, Santa Clara, CA, USA). Fluorescently labeled cRNA was generated using the Low Input QuickAmp Labeling Kit (Agilent Technologies, Santa Clara, CA, USA). Labeled cRNA was hybridized to the array using the Agilent Gene Expression Hybridization Kit (Agilent Technologies, Santa Clara, CA, USA). Arrays are washed twice prior to scanning with the SureScan Microarray Scanner (Agilent Technologies, Santa Clara, CA, USA). All statistical analysis and quality control of data was performed by OakLabs in order to generate a list of differentially expressed probes. Only probes which had a Log_2_fold change of greater than or less than 1 and a p-value of < .05 were considered. Heat maps displaying the z-score for differentially regulated genes were generated by OakLabs. Microarray data were submitted to the ArrayExpress database at EMBL-EBI under Accession Number E-MTAB-5966 (www.ebi.ac.uk/arrayexpress).

## Supporting information

S1 Fig In situ hybridization with sense probes for *b3glcta* and *b3glctb*. (TIF)

S2 Fig Heatmap including 483 differentially regulated transcripts shared between head and trunk tissues. (TIF)

S1 Table Summary of TALEN-generated genetic alleles and established lines. (DOCX)

S2 Table List of probes differentially regulated in both head and trunk tissues from *b3glct*^*-/-*^ embryos. (DOCX)

S3 Table Summary of PCR primers and assays. (DOCX)
