# Supplementary figures and images for: Functional characterization of zebrafish orthologs of the human Beta 3-Glucosyltransferase B3GLCT gene mutated in Peters Plus Syndrome
Source: PLoS One. 2017 Sep 19;12(9):e0184903. doi: 10.1371/journal.pone.0184903 (PMC5604996; doi:10.1371/journal.pone.0184903)

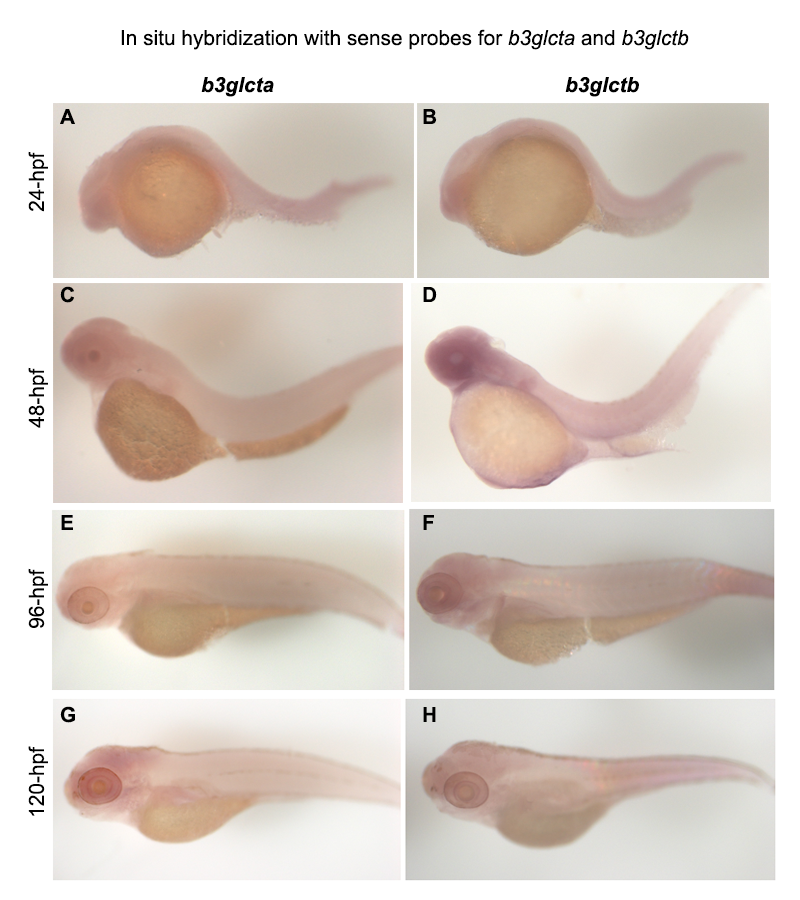

Supplement: S1 Fig — (TIF) [file pone.0184903.s001.tif]
