# Supplementary material for: Functional characterization of zebrafish orthologs of the human Beta 3-Glucosyltransferase B3GLCT gene mutated in Peters Plus Syndrome
Source: PLoS One. 2017 Sep 19;12(9):e0184903. doi: 10.1371/journal.pone.0184903 (PMC5604996; doi:10.1371/journal.pone.0184903)
Supplement: S1 Table — (DOCX) [file pone.0184903.s003.docx]

**Table S1.** Summary of TALEN-generated genetic alleles and established lines.

| **Allele #** | **Nucleotide** | **Protein** | **ZFIN Line Designation** |
| --- | --- | --- | --- |
| ***TALEN induced mutations in b3glcta exon 1*** | | | |
| 1 | c.24_25ins17 | p.(G9Sfs*9) |  |
| 2 | c.15_23del9 | p.(K5_Q7del) |  |
| 3 | c.15_19del5 | p.(C6Kfs*23) | mw707 |
| 4 | c.15_16delGT | p.(C6Sfs*24) | mw708 |
| 5 | c.14_15delAG | p.(K5Mfs*25) |  |
| 6 | c.5_17del13 | p.(S3Kfs*5) |  |
| 7 | c.15delG | p.(K5Nfs*7) |  |
| 8 | c.16_25del10 | p.(C6Dfs*3) |  |
| 9 | c.15_16dupGT | p.(Q7Vfs*6) |  |
| ***TALEN induced mutations in b3glcta exon 12*** | | | |
| 10 | c.1015delC | p.(H339Tfs*8) |  |
| 11 | c.1012_1017delAGTCAC | p.(S338_H339del) |  |
| 12 | c.1013delG | p.(S338Ifs*9) | mw705 |
| 13 | c.1013_1016delGTCA | p.(S338Tfs*8) |  |
| 14 | c.1012_15delAGTCinsCCAGA | p.(S338Pfs*6) |  |
| 15 | c.1009_1020del12 | p.(S337_V340del) |  |
| 16 | c.1011_1018delCAGTCACGinsAAC | p.(S337Rfs*5) |  |
| 17 | c.1014_1015insGTC | p.(S338_H339insV) |  |
| ***TALEN induced mutations in b3glctb exon 12*** | | | |
| 18 | c.1005_1026del22 | p.(A336Cfs*2) | mw706 |
