# Supplementary material for: Functional characterization of zebrafish orthologs of the human Beta 3-Glucosyltransferase B3GLCT gene mutated in Peters Plus Syndrome
Source: PLoS One. 2017 Sep 19;12(9):e0184903. doi: 10.1371/journal.pone.0184903 (PMC5604996; doi:10.1371/journal.pone.0184903)
Supplement: S2 Table — (DOCX) [file pone.0184903.s004.docx]

**Table S2**. List of probes differentially regulated in both head and trunk tissues from *b3glct^-/-^* embryos.

| **Fold Change *b3glct^-/-^* Head** | **Fold Change *b3glct^-/-^*Trunk** | **Log2FC *b3glct^-/-^* Head** | **Log2FC *b3glct^-/-^*Trunk** | **Zebrafish Transcript ID** | **Zebrafish Gene** | **Human Gene** |
| --- | --- | --- | --- | --- | --- | --- |
| 0.25 | 0.31 | -2.00 | -1.67 | ENSDART00000134559 | *aanat1* | *AANAT* |
| 0.43 | 0.40 | -1.21 | -1.34 | ENSDART00000011706 | *adprhl2* | *ADPRHL2* |
| 6.60 | 2.99 | 2.72 | 1.58 | ENSDART00000029781 | *ahsa1* | *AHSA1* |
| 6.62 | 3.20 | 2.73 | 1.68 | ENSDART00000132434 | *ahsa1a* | *AHSA1* |
| 9.85 | 3.16 | 3.30 | 1.66 | ENSDART00000153294 | *ahsa1a* | *AHSA1* |
| 0.49 | 0.33 | -1.04 | -1.62 | ENSDART00000131279 | *alg5* | *ALG5* |
| 0.50 | 0.32 | -1.01 | -1.63 | ENSDART00000140327 | *alg5* | *ALG5* |
| 0.24 | 0.27 | -2.04 | -1.89 | ENSDART00000109891 | *angel1* | *ANGEL1* |
| 0.37 | 0.35 | -1.45 | -1.53 | ENSDART00000079437 | *ap4m1* | *AP4M1* |
| 2.49 | 2.59 | 1.31 | 1.37 | ENSDART00000114915 | *aplnra* | *APLNR* |
| 11.34 | 13.81 | 3.50 | 3.79 | ENSDART00000138154 | *arhgap20b* | *ARHGAP20* |
| 3.57 | 4.15 | 1.84 | 2.05 | ENSDART00000134741 | *arhgef10lb* | *ARHGEF10L* |
| 0.20 | 0.27 | -2.35 | -1.91 | ENSDART00000090069 | *axdnd1* | *AXDND1* |
| 0.16 | 0.26 | -2.63 | -1.95 | ENSDART00000136908 | *axdnd1* | *AXDND1* |
| 0.42 | 0.33 | -1.26 | -1.59 | ENSDART00000113370 | *b3glcta* | *B3GLCT* |
| 2.79 | 2.78 | 1.48 | 1.47 | ENSDART00000099677 | *bloc1s3* | *BLOC1S3* |
| 3.10 | 2.63 | 1.63 | 1.39 | ENSDART00000156213 | *bloc1s3* | *BLOC1S3* |
| 0.21 | 0.20 | -2.27 | -2.35 | ENSDART00000109148 | *brap* | *BRAP* |
| 0.25 | 0.32 | -1.98 | -1.64 | ENSDART00000023262 | *C11orf88* | *C11orf88* |
| 2.43 | 2.61 | 1.28 | 1.39 | ENSDART00000062067 | *c14orf159* | *C14orf159* |
| 2.46 | 2.46 | 1.30 | 1.30 | ENSDART00000105562 | *C17orf64* | *C17orf62* |
| 0.20 | 0.15 | -2.31 | -2.72 | ENSDART00000087196 | *C17orf75* | *C17orf75* |
| 4.87 | 3.16 | 2.28 | 1.66 | ENSDART00000079709 | *c6* | *C6* |
| 7.48 | 4.57 | 2.90 | 2.19 | ENSDART00000134296 | *c6* | *C6* |
| 5.41 | 3.69 | 2.44 | 1.88 | ENSDART00000138155 | *c6* | *C6* |
| 7.55 | 4.69 | 2.92 | 2.23 | ENSDART00000139430 | *c6* | *C6* |
| 6.56 | 4.09 | 2.71 | 2.03 | ENSDART00000143988 | *c6* | *C6* |
| 6.72 | 4.06 | 2.75 | 2.02 | ENSDART00000146593 | *c6* | *C6* |
| 6.41 | 4.43 | 2.68 | 2.15 | ENSDART00000147988 | *c6* | *C6* |
| 0.30 | 0.44 | -1.73 | -1.18 | ENSDART00000154869 | *cacna1ha* | *CACNA1H* |
| 0.30 | 0.19 | -1.76 | -2.42 | ENSDART00000073778 | *caspb* | *CASP1* |
| 2.87 | 2.64 | 1.52 | 1.40 | ENSDART00000133171 | *cast* | *CAST* |
| 2.37 | 2.50 | 1.24 | 1.32 | ENSDART00000087148 | *cbln4* | *CBLN4* |
| 7.22 | 11.33 | 2.85 | 3.50 | ENSDART00000081385 | *ccdc172* | *CCDC172* |
| 3.35 | 2.72 | 1.75 | 1.44 | ENSDART00000156924 | *ccdc172* | *CCDC172* |
| 12.73 | 9.00 | 3.67 | 3.17 | ENSDART00000040598 | *cell* | *CEL* |
| 4.37 | 3.64 | 2.13 | 1.86 | ENSDART00000143258 | *cep19* | *CEP19* |
| 0.26 | 0.28 | -1.93 | -1.83 | ENSDART00000143074 | *clcn7* | *CLCN7* |
| 2.02 | 2.01 | 1.01 | 1.01 | ENSDART00000006173 | *cldn7b* | *CLDN7* |
| 0.14 | 0.12 | -2.82 | -3.05 | ENSDART00000146094 | *cldnf* | *CLDN3* |
| 0.00 | 0.00 | -8.54 | -7.89 | ENSDART00000145375 | *cpt1a* | *CPT1A* |
| 2.16 | 2.18 | 1.11 | 1.12 | ENSDART00000060347 | *crygs2* | *CRYGS* |
| 28.33 | 61.80 | 4.82 | 5.95 | ENSDART00000041877 | *csrnp1a* | *CSRNP1* |
| 0.34 | 0.30 | -1.56 | -1.76 | ENSDART00000006922 | *CTRL* | *CTRL* |
| 0.47 | 0.48 | -1.08 | -1.06 | ENSDART00000109851 | *CU570684.3* | *IGKV1-12* |
| 0.37 | 0.35 | -1.43 | -1.52 | ENSDART00000057528 | *cuedc2* | *CUEDC2* |
| 0.47 | 0.44 | -1.08 | -1.18 | ENSDART00000123482 | *cuedc2* | *CUEDC2* |
| 0.33 | 0.31 | -1.62 | -1.68 | ENSDART00000131609 | *cuedc2* | *CUEDC2* |
| 0.47 | 0.48 | -1.09 | -1.07 | ENSDART00000135265 | *cuedc2* | *CUEDC2* |
| 3.23 | 2.68 | 1.69 | 1.42 | ENSDART00000086534 | *cyb5d2* | *CYB5D2* |
| 2.92 | 2.87 | 1.55 | 1.52 | ENSDART00000122199 | *cyfip1* | *CYFIP1* |
| 5.83 | 3.43 | 2.54 | 1.78 | ENSDART00000122597 | *cyp2k19* | *CYP2W1* |
| 2.51 | 2.25 | 1.33 | 1.17 | ENSDART00000086197 | *dars2* | *DARS2* |
| 2.46 | 2.16 | 1.30 | 1.11 | ENSDART00000137170 | *dars2* | *DARS2* |
| 3.61 | 2.97 | 1.85 | 1.57 | ENSDART00000115136 | *dennd3a* | *DENND3* |
| 0.32 | 0.23 | -1.63 | -2.13 | ENSDART00000021864 | *dhrs13l1* | *DHRS13* |
| 0.28 | 0.22 | -1.84 | -2.17 | ENSDART00000112669 | *dhrs13l1* | *DHRS13* |
| 0.37 | 0.27 | -1.42 | -1.88 | ENSDART00000098963 | *dscama* | *DSCAM* |
| 0.36 | 0.37 | -1.48 | -1.45 | ENSDART00000125045 | *dscama* | *DSCAM* |
| 0.36 | 0.29 | -1.46 | -1.78 | ENSDART00000136928 | *dscama* | *DSCAM* |
| 4.22 | 2.34 | 2.08 | 1.23 | ENSDART00000123097 | *dusp27* | *DUSP27* |
| 2.41 | 2.70 | 1.27 | 1.43 | ENSDART00000125867 | *ECE2* | *ECE2* |
| 0.48 | 0.44 | -1.06 | -1.19 | ENSDART00000151939 | *eci2* | *ECI2* |
| 0.50 | 0.51 | -1.00 | -0.98 | ENSDART00000131676 | *eef2l2* | *EEF2* |
| 0.47 | 0.36 | -1.08 | -1.49 | ENSDART00000134098 | *efemp2a* | *EFEMP2* |
| 1.98 | 2.46 | 0.99 | 1.30 | ENSDART00000012717 | *ehd1b* | *EHD1* |
| 0.41 | 0.39 | -1.29 | -1.34 | ENSDART00000066645 | *eif1axa* | *EIF1AX* |
| 2.97 | 2.96 | 1.57 | 1.57 | ENSDART00000019053 | *faima* | *FAIM* |
| 4.04 | 3.98 | 2.01 | 1.99 | ENSDART00000084268 | *fam98b* | *FAM98B* |
| 0.36 | 0.28 | -1.49 | -1.82 | ENSDART00000045135 | *fbxo16* | *FBXO16* |
| 0.26 | 0.33 | -1.96 | -1.61 | ENSDART00000153098 | *fbxo16* | *FBXO16* |
| 0.38 | 0.37 | -1.41 | -1.43 | ENSDART00000018144 | *fgd4a* | *FGD4* |
| 0.41 | 0.39 | -1.30 | -1.36 | ENSDART00000051606 | *ficd* | *FICD* |
| 0.35 | 0.24 | -1.51 | -2.04 | ENSDART00000110902 | *frmpd1b* | *FRMPD1* |
| 3.23 | 3.79 | 1.69 | 1.92 | ENSDART00000143482 | *fsd1l* | *FSD1L* |
| 0.25 | 0.24 | -2.02 | -2.09 | ENSDART00000154890 | *ganc* | *GANC* |
| 2.22 | 2.21 | 1.15 | 1.14 | ENSDART00000055965 | *gcdhl* | *GCDH* |
| 2.21 | 2.67 | 1.15 | 1.42 | ENSDART00000080430 | *gfra2b* | *GFRA2* |
| 3.00 | 2.73 | 1.58 | 1.45 | ENSDART00000063781 | *gpr55a* | *GPR55* |
| 2.15 | 2.34 | 1.11 | 1.23 | ENSDART00000021437 | *gria1a* | *GRIA1* |
| 0.37 | 0.42 | -1.43 | -1.24 | ENSDART00000044080 | *her12* | *HES5* |
| 1.98 | 2.37 | 0.99 | 1.25 | ENSDART00000037906 | *hirip3* | *HIRIP3* |
| 4.24 | 4.27 | 2.08 | 2.09 | ENSDART00000151468 | *hivep1* | *HIVEP1* |
| 0.18 | 0.16 | -2.46 | -2.67 | ENSDART00000059690 | *hps3* | *HPS3* |
| 0.19 | 0.15 | -2.39 | -2.69 | ENSDART00000140968 | *hps3* | *HPS3* |
| 0.45 | 0.44 | -1.14 | -1.17 | ENSDART00000048759 | *id2b* | *ID2* |
| 0.32 | 0.29 | -1.65 | -1.79 | ENSDART00000057865 | *ier3ip1* | *IER3IP1* |
| 0.28 | 0.17 | -1.86 | -2.56 | ENSDART00000135941 | *il11ra* | *IL11RA* |
| 2.01 | 2.21 | 1.00 | 1.14 | ENSDART00000009932 | *ints6* | *INTS6* |
| 0.44 | 0.41 | -1.20 | -1.27 | ENSDART00000078796 | *kansl2* | *KANSL2* |
| 0.42 | 0.39 | -1.24 | -1.36 | ENSDART00000146332 | *kansl2* | *KANSL2* |
| 0.37 | 0.34 | -1.42 | -1.56 | ENSDART00000146720 | *lin7c* | *LIN7C* |
| 2.19 | 2.42 | 1.13 | 1.28 | ENSDART00000020819 | *lman2l* | *LMAN2L* |
| 2.68 | 2.31 | 1.42 | 1.21 | ENSDART00000022170 | *LTN1* | *LTN1* |
| 0.24 | 5.04 | -2.08 | 2.33 | ENSDART00000109231 | *mamdc2b* | *MAMDC2* |
| 2.81 | 2.14 | 1.49 | 1.10 | ENSDART00000113243 | *manba* | *MANBA* |
| 0.34 | 0.35 | -1.56 | -1.51 | ENSDART00000154432 | *masp1* | *MASP1* |
| 0.11 | 0.28 | -3.25 | -1.84 | ENSDART00000113521 | *matk* | *MATK* |
| 0.21 | 0.19 | -2.24 | -2.43 | ENSDART00000075218 | *me1* | *ME1* |
| 0.20 | 0.20 | -2.32 | -2.34 | ENSDART00000101969 | *me1* | *ME1* |
| 0.21 | 0.20 | -2.24 | -2.31 | ENSDART00000110493 | *ME1* | *ME1* |
| 0.22 | 0.21 | -2.17 | -2.25 | ENSDART00000147941 | *me1* | *ME1* |
| 7.56 | 8.23 | 2.92 | 3.04 | ENSDART00000066154 | *med11* | *MED11* |
| 6.46 | 4.30 | 2.69 | 2.10 | ENSDART00000140995 | *mfsd2b* | *MFSD2B* |
| 0.24 | 0.18 | -2.03 | -2.51 | ENSDART00000134062 | *mhc1zja* | *FCGRT* |
| 0.47 | 0.48 | -1.08 | -1.06 | ENSDART00000133555 | *mical3a* | *MICAL3* |
| 0.43 | 0.43 | -1.22 | -1.22 | ENSDART00000147613 | *mical3a* | *MICAL3* |
| 0.36 | 0.29 | -1.49 | -1.79 | ENSDART00000105427 | *mif* | *MIF* |
| 2.43 | 3.10 | 1.28 | 1.63 | ENSDART00000135583 | *mlpha* | *MLPH* |
| 0.38 | 0.39 | -1.41 | -1.37 | ENSDART00000146041 | *mthfsd* | *MTHFSD* |
| 2.63 | 2.69 | 1.39 | 1.43 | ENSDART00000052422 | *mtx1b* | *MTX1* |
| 0.50 | 0.49 | -0.99 | -1.03 | ENSDART00000040577 | *mybl2* | *MYBL2* |
| 0.50 | 0.49 | -1.00 | -1.04 | ENSDART00000007914 | *nabp1a* | *NABP1* |
| 0.47 | 0.44 | -1.08 | -1.19 | ENSDART00000135356 | *nabp1a* | *NABP1* |
| 0.30 | 0.24 | -1.73 | -2.09 | ENSDART00000135221 | *NEPRO* | *NEPRO* |
| 0.36 | 0.33 | -1.49 | -1.58 | ENSDART00000151951 | *nlrb5* | *NLRP1* |
| 0.44 | 0.43 | -1.17 | -1.21 | ENSDART00000099799 | *nrip1b* | *NRIP1* |
| 2.03 | 3.05 | 1.02 | 1.61 | ENSDART00000145721 | *nrn1la* | *NRN1L* |
| 0.36 | 0.35 | -1.47 | -1.51 | ENSDART00000065125 | *nt5c2b* | *NT5C2* |
| 0.34 | 0.31 | -1.55 | -1.71 | ENSDART00000152232 | *nt5c2b* | *NT5C2* |
| 0.38 | 0.34 | -1.40 | -1.56 | ENSDART00000152493 | *nt5c2b* | *NT5C2* |
| 0.46 | 0.42 | -1.11 | -1.26 | ENSDART00000016976 | *nucks1b* | *NUCKS1* |
| 0.41 | 0.41 | -1.29 | -1.30 | ENSDART00000051224 | *pafah1b2* | *PAFAH1B2* |
| 7.00 | 8.17 | 2.81 | 3.03 | ENSDART00000027212 | *PARP9* | *PARP9* |
| 0.18 | 0.17 | -2.45 | -2.55 | ENSDART00000123667 | *pkma* | *PKM* |
| 3.90 | 3.40 | 1.96 | 1.77 | ENSDART00000067596 | *PLA2G4C* | *PLA2G4C* |
| 0.30 | 0.26 | -1.74 | -1.95 | ENSDART00000054681 | *pm20d1.1* | *PM20D1* |
| 0.30 | 0.24 | -1.73 | -2.09 | ENSDART00000136967 | *pm20d1.1* | *PM20D1* |
| 0.35 | 0.27 | -1.52 | -1.90 | ENSDART00000139695 | *pm20d1.1* | *PM20D1* |
| 0.29 | 0.37 | -1.77 | -1.44 | ENSDART00000149898 | *pnp5b* | *PNP* |
| 0.33 | 0.40 | -1.58 | -1.33 | ENSDART00000145791 | *prune2* | *PRUNE2* |
| 0.48 | 0.45 | -1.06 | -1.14 | ENSDART00000039822 | *ptbp1b* | *PTBP1* |
| 0.07 | 0.08 | -3.89 | -3.67 | ENSDART00000014047 | *pth2r* | *PTH2R* |
| 0.39 | 0.49 | -1.35 | -1.03 | ENSDART00000110037 | *ptprb* | *PTPRB* |
| 0.36 | 0.27 | -1.46 | -1.88 | ENSDART00000092609 | *pvrl2l* | *PVRL2* |
| 0.40 | 0.38 | -1.33 | -1.40 | ENSDART00000122870 | *RAB11FIP5* | *RAB11FIP5* |
| 3.24 | 3.11 | 1.70 | 1.64 | ENSDART00000060321 | *RAMP1* | *RAMP1* |
| 0.33 | 0.28 | -1.61 | -1.86 | ENSDART00000136855 | *rca2.2* | *CD46* |
| 2.34 | 2.55 | 1.23 | 1.35 | ENSDART00000138953 | *rgcc* | *RGCC* |
| 2.34 | 3.97 | 1.23 | 1.99 | ENSDART00000077166 | *rgra* | *RGR* |
| 2.85 | 2.85 | 1.51 | 1.51 | ENSDART00000063434 | *ripk4* | *RIPK4* |
| 2.51 | 2.75 | 1.33 | 1.46 | ENSDART00000128181 | *ripk4* | *RIPK4* |
| 3.11 | 3.29 | 1.64 | 1.72 | ENSDART00000131291 | *ripk4* | *RIPK4* |
| 2.00 | 2.35 | 1.00 | 1.23 | ENSDART00000026694 | *rpp40* | *RPP40* |
| 0.28 | 0.50 | -1.84 | -1.00 | ENSDART00000058459 | *rspo1* | *RSPO1* |
| 0.49 | 0.36 | -1.02 | -1.48 | ENSDART00000076749 | *samsn1a* | *SAMSN1* |
| 0.49 | 0.44 | -1.04 | -1.20 | ENSDART00000099947 | *samsn1a* | *SAMSN1* |
| 2.55 | 2.47 | 1.35 | 1.30 | ENSDART00000021736 | *scocb* | *SCOC* |
| 0.34 | 0.31 | -1.54 | -1.71 | ENSDART00000137706 | *scrn3* | *SCRN3* |
| 0.15 | 0.13 | -2.78 | -2.92 | ENSDART00000109752 | *serpinh1a* | *SERPINH1* |
| 6.61 | 2.10 | 2.73 | 1.07 | ENSDART00000141476 | *sgms2* | *SGMS2* |
| 0.18 | 0.17 | -2.45 | -2.60 | ENSDART00000155804 | *si:ch1073-185p12.2* | *GIMAP1-GIMAP5* |
| 11.90 | 13.13 | 3.57 | 3.71 | ENSDART00000111180 | *si:ch211-186e20.2* | *SERPINA1* |
| 10.10 | 4.58 | 3.34 | 2.20 | ENSDART00000149850 | *si:ch211-186e20.2* | *SERPINA4* |
| 4.44 | 3.62 | 2.15 | 1.86 | ENSDART00000051469 | *si:ch211-186e20.7* | *SERPIN* |
| 7.00 | 2.45 | 2.81 | 1.30 | ENSDART00000149066 | *si:ch211-186e20.7* | *SERPINA4* |
| 4.60 | 3.73 | 2.20 | 1.90 | ENSDART00000142267 | *si:ch211-197g15.10* | *IFI44* |
| 3.63 | 3.67 | 1.86 | 1.88 | ENSDART00000105460 | *si:ch211-197g15.6* | *IFI44* |
| 2.66 | 2.76 | 1.41 | 1.46 | ENSDART00000139658 | *si:ch211-197g15.6* | *IFI44L* |
| 0.43 | 0.22 | -1.23 | -2.15 | ENSDART00000113134 | *si:ch211-233g6.8* | *CASP* |
| 0.32 | 0.38 | -1.65 | -1.40 | ENSDART00000132376 | *si:dkey-27p23.3* | *C11orf88* |
| 0.49 | 0.31 | -1.03 | -1.68 | ENSDART00000140829 | *si:dkey-78l4.10* | *GZMM* |
| 1.96 | 2.24 | 0.97 | 1.16 | ENSDART00000040793 | *sirt5* | *SIRT5* |
| 0.37 | 0.44 | -1.44 | -1.17 | ENSDART00000057584 | *slc1a4* | *SLC1A4* |
| 0.19 | 0.09 | -2.41 | -3.46 | ENSDART00000144003 | *slc23a3* | *SLC23A3* |
| 3.44 | 4.58 | 1.78 | 2.20 | ENSDART00000139156 | *slc37a4b* | *SLC37A4* |
| 2.38 | 3.00 | 1.25 | 1.58 | ENSDART00000066631 | *slc51a* | *SLC51A* |
| 3.78 | 2.48 | 1.92 | 1.31 | ENSDART00000045423 | *slc7a2* | *SLC7A2* |
| 3.77 | 2.11 | 1.91 | 1.08 | ENSDART00000126009 | *slc7a2* | *SLC7A2* |
| 9.40 | 10.90 | 3.23 | 3.45 | ENSDART00000118868 | *snoU13* | *snoU13* |
| 0.41 | 0.37 | -1.29 | -1.45 | ENSDART00000151868 | *spty2d1* | *SPTY2D1* |
| 0.29 | 0.33 | -1.78 | -1.61 | ENSDART00000142167 | *st6gal2a* | *ST6GAL2* |
| 4.09 | 5.20 | 2.03 | 2.38 | ENSDART00000011568 | *syngr3a* | *SYNGR3* |
| 2.16 | 2.36 | 1.11 | 1.24 | ENSDART00000129501 | *TMEM235* | *TMEM235* |
| 3.35 | 3.60 | 1.75 | 1.85 | ENSDART00000127689 | *TMEM245* | *TMEM245* |
| 2.86 | 3.90 | 1.52 | 1.96 | ENSDART00000144459 | *trim35-22* | *TRIM35* |
| 2.50 | 2.79 | 1.32 | 1.48 | ENSDART00000057464 | *trim35-31* | *TRIM35* |
| 5.99 | 6.44 | 2.58 | 2.69 | ENSDART00000086144 | *trim35-33* | *TRIM35* |
| 2.30 | 2.10 | 1.20 | 1.07 | ENSDART00000146662 | *trim35-33* | *TRIM35* |
| 3.26 | 2.55 | 1.71 | 1.35 | ENSDART00000140241 | *trim35-37* | *TRIM35* |
| 2.35 | 2.61 | 1.23 | 1.39 | ENSDART00000112581 | *trmt10c* | *TRMT10C* |
| 2.17 | 2.28 | 1.12 | 1.19 | ENSDART00000144606 | *trmt10c* | *TRMT10C* |
| 3.66 | 2.71 | 1.87 | 1.44 | ENSDART00000105898 | *TSTD1* | *TSTD1* |
| 0.39 | 0.25 | -1.36 | -2.02 | ENSDART00000061838 | *ttc38* | *TTC38* |
| 2.19 | 2.02 | 1.13 | 1.01 | ENSDART00000115842 | *u2* | *RNU2-14P* |
| 2.19 | 2.07 | 1.13 | 1.05 | ENSDART00000118301 | *u2* | *RNU2-14P* |
| 2.20 | 2.01 | 1.14 | 1.01 | ENSDART00000119007 | *u2* | *RNU2-14P* |
| 5.13 | 4.27 | 2.36 | 2.10 | ENSDART00000014649 | *ugt2a5* | *UGT2B10* |
| 2.65 | 2.82 | 1.40 | 1.50 | ENSDART00000034620 | *ugt2a6* | *UGT2BA3* |
| 2.88 | 3.18 | 1.52 | 1.67 | ENSDART00000073663 | *ugt2a6* | *UGT2A1* |
| 2.53 | 2.48 | 1.34 | 1.31 | ENSDART00000154390 | *ugt2a6* | *UGT2A1* |
| 2.91 | 2.51 | 1.54 | 1.33 | ENSDART00000136333 | *wash* | *WASH1* |
| 0.34 | 0.50 | -1.54 | -1.00 | ENSDART00000108635 | *wdr27* | *WDR27* |
| 0.50 | 0.38 | -1.01 | -1.40 | ENSDART00000133479 | *zgc:153901* | *FTCDNL1* |
| 11.80 | 7.40 | 3.56 | 2.89 | ENSDART00000149563 | *zgc:174259* | *SERPINA12* |
| 8.26 | 3.59 | 3.05 | 1.85 | ENSDART00000007636 | *zgc:77118* | *PPT2* |
| 0.32 | 0.31 | -1.64 | -1.70 | ENSDART00000024558 | *zgc:92137* | *AMY1A* |
| 0.45 | 0.44 | -1.15 | -1.17 | ENSDART00000076800 | *znhit1* | *ZNHIT1* |
