# Supplementary material for: Functional characterization of zebrafish orthologs of the human Beta 3-Glucosyltransferase B3GLCT gene mutated in Peters Plus Syndrome
Source: PLoS One. 2017 Sep 19;12(9):e0184903. doi: 10.1371/journal.pone.0184903 (PMC5604996; doi:10.1371/journal.pone.0184903)
Supplement: S3 Table — (DOCX) [file pone.0184903.s005.docx]

**Table S3.** Summary of PCR primers and assays.

| **Oligonucleotide** | **Forward** | **Reverse** | **Product size** |
| --- | --- | --- | --- |
| ***RACE*** | | | |
| *b3glcta* RACE | ACAGCACACATAGGATACACAGAGC |  |  |
| *b3glcta*_3´ |  | TCATACAGACCAGAAGGCAC | 1678 bp (with *b3glcta_RACE*) |
| *b3glcta*_nested |  | GTCTGACAGCGTATGCAGGA | 347 bp (with *b3glcta_RACE*) |
| *b3glctb* RACE | TTTGTGCTCCATGTTTCTGC |  |  |
| *b3glctb*_3´ |  | GCAGCATCTTTTTCCCATGT | 939 bp (with *b3glctb_RACE*) |
| *b3glctb*_nested |  | CAGTCTGAGCCTGATGGAGAA | 302 bp (with *b3glctb_RACE*) |
| ***RT-PCR*** | | | |
| *b3glcta* | CGCTTTCGAGTACCCAGACT | CTTTGCCATCCTCCCAGATA | 155 bp |
| *b3glctb* | GCGGGATGTGTTATTTGTGA | CGGAAGAATGCTCCAGTTTC | 172 bp |
| *rhodopsin* | CGGCCTACATGTTCTTCCTC | GGTGTACATGGTGGTGGTGA | 170 bp |
| *pitx2c* | GATGTGCAGGAGAGTGTGTGT | TCTGGACTGGAGGTGTCTGA | 543 bp |
| *b-actin* | GAGAAGATCTGGCATCACAC | ATCAGGTAGTCTGTCAGGTC | 324 bp |
| ***In-situ hybridization probe*** | | | |
| *b3glcta* | CTGCATACGCTGTCAGACAA | CTATAATTCCTCCTTAGTGC | 1209 bp |
| *b3glctb* | GCTGAAGGAGGACGTCTTTG | TACAGCTCCTCTCTGGTGTC | 696 bp |
| ***PCR primers to verify TALEN knockout and genotype zebrafish lines*** | | | |
| *b3glcta-exon 1* | ATGTCAAGCGACAAGCACTG | GGCAGCACAGTCATTCATTC | 663 bp |
| *b3glcta-exon12* | TGTGAACGTTTCACGATTTTG | CATACCGCTCTCCCAGACAC | 400 bp |
| *b3glctb-exon12* | CACAGTCCAGTGGCTTGTCT | TGGATGAGTGTTGGCCTGAC | 223 bp |
